# Clinical Importance of Steps Taken per Day among Persons with Multiple Sclerosis

**DOI:** 10.1371/journal.pone.0073247

**Published:** 2013-09-04

**Authors:** Robert W. Motl, Lara A. Pilutti, Yvonne C. Learmonth, Myla D. Goldman, Ted Brown

**Affiliations:** 1 Department of Kinesiology and Community Health, University of Illinois at Urbana-Champaign, Urbana, Illinois, United States of America; 2 Department of Neurology, University of Virginia, Charlottesville, Virginia, United States of America; 3 MS Center at Evergreen, Evergreen Health, Evergreen, Washington, United States of America; Institute Biomedical Research August Pi Sunyer (IDIBAPS) – Hospital Clinic of Barcelona, Spain

## Abstract

**Background:**

The number of steps taken per day (steps/day) provides a reliable and valid outcome of free-living walking behavior in persons with multiple sclerosis (MS).

**Objective:**

This study examined the clinical meaningfulness of steps/day using the minimal clinically important difference (MCID) value across stages representing the developing impact of MS.

**Methods:**

This study was a secondary analysis of de-identified data from 15 investigations totaling 786 persons with MS and 157 healthy controls. All participants provided demographic information and wore an accelerometer or pedometer during the waking hours of a 7-day period. Those with MS further provided real-life, health, and clinical information and completed the Multiple Sclerosis Walking Scale-12 (MSWS-12) and Patient Determined Disease Steps (PDDS) scale. MCID estimates were based on regression analyses and analysis of variance for between group differences.

**Results:**

The mean MCID from self-report scales that capture subtle changes in ambulation (1-point change in PDSS scores and 10-point change in MSWS-12 scores) was 779 steps/day (14% of mean score for MS sample); the mean MCID for clinical/health outcomes (MS type, duration, weight status) was 1,455 steps/day (26% of mean score for MS sample); real-life anchors (unemployment, divorce, assistive device use) resulted in a mean MCID of 2,580 steps/day (45% of mean score for MS sample); and the MCID for the cumulative impact of MS (MS vs. control) was 2,747 steps/day (48% of mean score for MS sample).

**Conclusion:**

The change in motion sensor output of ∼800 steps/day appears to represent a lower-bound estimate of clinically meaningful change in free-living walking behavior in interventions of MS.

## Introduction

There has been an ongoing debate regarding outcome measures in clinical research involving persons with multiple sclerosis (MS) [1], with increasing interest in approaches for objectively monitoring patients under real-world conditions [2]. This interest has highlighted the potential for the objective monitoring of free-living walking behavior using motion sensors such as accelerometers and pedometers in clinical research involving persons with neurologic diseases [3] including MS [2]. Such devices are worn around the waist or ankle during the waking hours of the day and over a representative sampling period (e.g., seven days). The motion sensors capture the total amount of walking undertaken in free-living conditions based on metrics such as steps taken per day (steps/day). The number of steps/day reflects a straight-forward metric of the overall amount of walking undertaken during one's everyday life, representing free-living walking behavior [2], [3].

Accumulating data demonstrates that the number of steps/day provides a reliable and valid measure of free-living walking behavior in MS [4]–[7]. Steps/day has demonstrated acceptable test-retest reliability over a two-week time period in persons with MS [4], and as few as three days of data with an appropriate amount of wear time (i.e., 10 or more hours/day) yields a reliable estimate of usual ambulatory-based behavior [5]. Regarding validity, steps/day has correlated strongly with clinical (e.g., Expanded Disability Status Scale scores), performance (e.g., timed 25-foot walk and 6-minute walk), and patient-reported (e.g., 12-Item Multiple Sclerosis Walking Scale or MSWS-12 scores) measures of ambulation in persons with MS [6], [7]. To date, there are no published data evaluating the clinical importance of differences in steps/day among those with MS (i.e., amount of difference in steps/day that actually reflects a difference) on a group level. Such minimal clinically important difference (MCID) values are necessary for designing and interpreting clinical trials wherein steps/day is an outcome measure of free-living walking behavior.

There are many possible approaches for establishing MCID values. We a priori propose an approach wherein MCID values are generated based on stages corresponding with the developing impact of MS. This would involve, for example, stages of MCID values based on smaller changes through to large changes in the impact of MS, progressively indicated by (a) scales that capture subtle, perceived changes in ambulation (e.g., MSWS-12 and Patient Determined Disease Steps or PDDS scale); (b) clinical and health outcomes including MS type, disease duration, and overweight/obesity status based on body mass index (BMI); (c) real-life anchors including unemployment, divorce, and assistive device requirements (e.g., cane for ambulation); and (d) cumulative impact of MS based on a comparison with healthy controls. Such an approach could provide several vantage points of the MCID, including stages and ranges of MCIDs, and afford researchers options for MCID selection that are appropriate for specific populations and circumstances (e.g., overall disease activity vs. symptomatic changes) in MS interventions.

The present study involved a secondary analysis of a combined data set from multiple investigations for establishing the clinical importance of steps/day in persons with MS based on stages that correspond with the developing impact of MS. We hypothesized that the MCID values would increase as a function of expressing the degree of impact of MS, moving from subtle, perceived differences in ambulation through to a comparison with healthy controls that represents the cumulative disease impact. The primary end result of the secondary analysis of data involved establishing stages and ranges of MCID estimates for interpreting changes in steps/day among persons with MS.

## Methods

### Ethics statement

The studies and associated procedures were all approved by the Institutional Review Board on the University of Illinois at Urbana-Champaign campus, and all participants provided written informed consent. The informed consent across all studies was broadly for examinations of ambulatory physical activity behavior and its determinants and consequences in persons with MS and/or healthy controls.

### Participants

This secondary analysis was performed on a combined dataset of persons with MS and healthy controls from a series of previous investigations of physical activity and its associations with symptomatic, social cognitive, or quality of life outcomes. The data from each investigation had been de-identified before amalgamation and analysis as a combined dataset in this paper; this precluded examination of seasonal effects as there was no link between data and identifications, but the data were collected across all seasons over a multi-year period. Participants with MS were recruited from throughout the United States using print and email flyers and an online advertisement on the National Multiple Sclerosis Society website. Healthy controls were recruited via public e-mail postings delivered across a University community and “word-of-mouth.” The common inclusion criteria across the investigations for persons with MS were (a) diagnosis of MS (both self-reported and neurologist confirmed), (b) relapse free in the previous 30 days, (c) ambulatory with or without assistive devices, (d) age between 18 and 64 years, and (e) willingness to wear a motion sensor for 7 days. The same inclusion criteria were applied for the healthy controls, with the exception of diagnosis of MS and relapse free over the past 30 days. The final combined samples included 786 persons with MS and 157 healthy controls, and all persons satisfied inclusion criteria and provided usable steps/day data for analyses (i.e., 3 or more days of data with sufficient wear time for generating a reliable estimate of usual behavior) [5].

### Devices

Steps/day were measured with Yamax SW-200 (NEWlifestyles) pedometers or ActiGraph accelerometers (models 7164 or GT3X; ActiGraph), as there is evidence for the accuracy of these devices during normal walking speeds in persons with MS and controls [8], [9]. For example, there was ∼4% or less error in the accuracy of ActiGraph accelerometers for measuring steps taken across speeds ranging between 54 and 107 m/min under controlled conditions in 24 adults with MS and 24 healthy controls [9]. There similarly was ∼4% or less error in the accuracy of Yamax pedometers for measuring steps taken across speeds ranging between 67 and 94 m/min under controlled conditions in 23 adults with MS [8]. The data were collected using 50 SW-200 pedometers, 100 model 7164 accelerometers, and 50 model GT3X accelerometers. All devices were calibrated for measuring 500 steps taken by laboratory staff members while walking on a treadmill at 3 mph and 0% gradient prior to use in the research projects. This calibration was undertaken to minimize variation among devices and inaccuracy as sources of error in the study outcome. Multiple types of devices were included as there was insufficient quantity of any one device for systematically capturing the free-living walking behavior across all participants. This further facilitated a comparison of steps/day between pedometer and accelerometer devices as accelerometers might be more accurate than pedometers.

The Yamax SW-200 pedometer measures steps over time using a spring-loaded lever arm and digital counter that displays steps/day. The actual reading on the pedometer was recorded by the participants in a log, and the device was reset daily. There was no method of validating wear time across individual days using the pedometer. The ActiGraph accelerometer (models 7164 and GT3X) measures steps using a piezoelectric bender element that produces an electric signal proportionate to the force acting upon it during movement. The steps were recorded over one-minute intervals, stored in the accelerometer's memory, and then downloaded by the research team on a personal computer. Steps per one-minute interval were later summed over the course of the day into steps/day. Accelerometer data were checked against participant recorded wear times from the log sheet and only valid days (≥10 hours of wear time without periods of continuous zeros exceeding 60 minutes) were included in the analysis. The outcome of both devices was steps/day averaged over 3 or more available days of data with sufficient wear time. There is evidence that 3 of more days of steps/day data provides a reliable estimate of usual walking behavior in persons with MS (intraclass correlation ≥.80) [5] and many researchers have included only a single day of valid data for measuring usual ambulatory-based activity. Accordingly, the analysis included persons who had 3 or more days of steps/day data.

### Walking impairment

The MSWS-12 is a 12-item patient-rated measure of the impact of MS on walking-related activities (including walking, running, standing, climbing stairs) [10]. The items are rated on a 5-point scale of 1 (*Not at all*) to 5 (*Extremely*), and the items represent limitations of walking during the past 2 weeks. The MSWS-12 was scored as recommended [10] and resulted in a total score that ranged between 0 and 100. The MSWS-12 has good evidence for its reliability and validity of scores as a measure of walking impairment in MS [10]. We examined values of steps/day across 10-point increments of MSWS-12 scores as this value reflects equivalent incremental changes across the entire range of MSWS-12 scores.

### Disability status

The Patient-Determined Disease Steps (PDDS) scale is a single item for measuring self-reported disability status on an ordinal scale ranging from 0 (*Normal*) through 8 (*Bedridden*) [11]. This scale was developed as an alternate for the physician-rated Expanded Disability Status Scale (EDSS), and scores from the PDDS have been linearly and strongly related with EDSS scores derived from a neurological examination [12]. Persons were categorized into no device, cane use, and bilateral support for ambulation based on PDDS scale scores of 1–3, 4–5, and 6, respectively, for analyses. We examined steps/day across 1-point incremental increases in PDDS scores and this is consistent with 1-point changes in EDSS considered as reflecting worsening MS disability.

### Socio-demographic, real-life, and health variables

We included a standard scale to measure all sociodemographic, health, and real-life variables. The sociodemographic variables included sex, age, race, and education for descriptive purposes. The scale further included marital status (coded as married vs. divorced for analyses with all other categories excluded), height and weight for generation of body mass index (BMI; coded as normal weight, overweight, and obese based on recommended guidelines of 18.5–24.9, 25.0–29.9, and ≥30.0 kg/m^2^, respectively, for analyses) [13], and employment (coded as employed vs. unemployed for analyses) for analyses.

### Clinical variables

We included a standard scale for measuring clinical variables in those with MS. Clinical course of MS was recorded based on descriptions provided in Lublin and Reingold [14] and coded for analyses as relapsing-remitting or progressive forms of MS. Disease duration was recorded based on time since the date of confirmed MS diagnosis and coded for analyses as early (<10 years), middle (10–20 years), or later (>20 years) stage of MS.

### Procedure

The studies and associated procedures were all approved by the same University institutional review board, and all participants provided written informed consent. The studies were conducted during all seasons of the year over a three-year period. After telephone screening for inclusion and provision of a signed informed consent document, all participants received a pedometer or accelerometer, a log sheet, instructions for wearing the device, and the scale for measuring sociodemographic, real-life, and health outcomes; those with MS further received the PDDS scale, MSWS-12, and measure of clinical outcomes. Participants were provided with written and graphical instructions to wear the pedometer or accelerometer on the provided belt around the waist over the non-dominant hip during all waking hours of a 7-day period, except when swimming, bathing, or showering; the 7-day period is standard in applications of motion sensors for measuring ambulatory activity. Waking hours were defined for participants as the moment of getting out of bed in the morning until the moment of getting into bed in the evening. During this one-week period, participants were asked to maintain normal routines and usual levels of physical activity. Participants were provided with a log sheet to record the time of day that the unit was worn and any times throughout the day that the unit was not worn. Those who received pedometers further recorded steps/day on the log before resetting the unit on a daily basis. Participants returned the study materials after the one-week wear time.

### Data analysis

All analyses were performed on de-identified data in PASW 18.0 (SPSS, Inc., Chicago, IL). Only participants with three or more days of steps/day data with acceptable wear time were included in the analysis (*N*s = 786 and 157 for MS and healthy controls, respectively). There were 31 persons with MS (i.e., 3.8% of the original MS sample of 817 who volunteered) and 14 controls (i.e., 8.2% of the original control sample of 171 who volunteered) without sufficient wear time for inclusion of the data in the analyses (i.e., <3 days of data). Descriptive statistics are presented in text and tables as mean (*M*) ± standard deviation (*SD*) along with 95% confidence interval (95% *CI*) unless otherwise noted (e.g., median and range or percentage). We first conducted a linear regression by regressing steps/day on PDDS and MSWS-12 scores, respectively, for estimating the incremental change in steps/day per unit change in walking impairment or disability (i.e., subtle, perceived MS changes). The remaining analyses all involved between-subjects analysis of variance (ANOVA) on steps/day. The initial set of analyses with ANOVA compared steps/day between clinical and health outcomes including MS type, disease duration, and overweight/obesity status based on BMI categories. The next set of analyses with ANOVA compared steps/day between real-life anchors including unemployment, divorce, and assistive device requirements. The last ANOVA examined the cumulative impact of MS based on a comparison between MS and healthy controls. The ANOVAs involved Bonferonni follow-ups that identified specific differences in analyses with three levels of a between-subjects factor and provided mean differences in steps/day between groups along with 95% *CI*. We adopted an alpha value of .01 when judging statistical significance to control the study-wise error rate. The standardized effect size (Cohen's d) was estimated as the difference in means between groups divided by the pooled standard deviation [15]. The data are available from the first author upon formal written request and approval of the University of Illinois.

## Results

### Demographic and clinical characteristics of samples

The demographic characteristics along with identified differences between the samples of persons with MS and healthy controls are provided in Table 1. The table further contains the clinical characteristics of the persons with MS. The median PDDS score was 2.0 (range = 0–6). This indicated that the sample overall was characterized by moderate disability (i.e., no limitations in walking but significant problems due to MS that limit daily activity in other ways) with a range between normal and two-point assistance (e.g., rollator or frame). The average duration of MS was 9.9 years with nearly 90% (*n* = 710) of the sample reporting a diagnosis of relapsing-remitting multiple sclerosis (RRMS). We do not have data for characterizing the current treatment regimens and mental status of the patients in this sample, and the PDDS has not been validated for capturing the Multiple Sclerosis Severity Score as a marker of disease severity.

**Table 1:** Demographic and clinical characteristics of the samples of multiple sclerosis and healthy controls.

Variable	Group		
	MS (*N* = 786)	Controls (*N* = 157)	*p*-value
Age (years)	47.3 (10.5)	43.4 (9.8)	.0001
Height (cm)	167.8 (9.3)	167.8 (8.2)	.959
Weight (kg)	78.6 (20.4)	73.8 (16.0)	.006
BMI (kg/m^2^)	27.9 (7.6)	26.2 (5.7)	.007
Sex (% female/male)	84.9/15.1	91.1/8.9	.035
Education (% college graduate)	21.7	51.2	.0001
Income (% >$40,000 year)	68.3	80.7	.001
Employment (% employed)	62.3	94.6	.0001
Race (% Caucasian)	92.0	79.5	.0001
MS Type (% RRMS)	89.7	–	
Disease duration (years)	9.9 (8.0)	–	
PDDS score (mdn, IQR)	2.0 (2.0)	–	
MSWS-12	40.3 (29.1)	–	

*Note*. Values are mean (standard deviation), unless otherwise noted. MS  =  multiple sclerosis. BMI  =  body mass index. RRMS  =  relapsing-remitting multiple sclerosis. PDDS  =  Patient Determined Disease Steps scale; MSWS-12  =  Multiple Sclerosis Walking Scale-12.

### Regression analyses

#### Steps/day Based on Perceived Changes in Ambulation of Persons with MS

**Figure 1:**
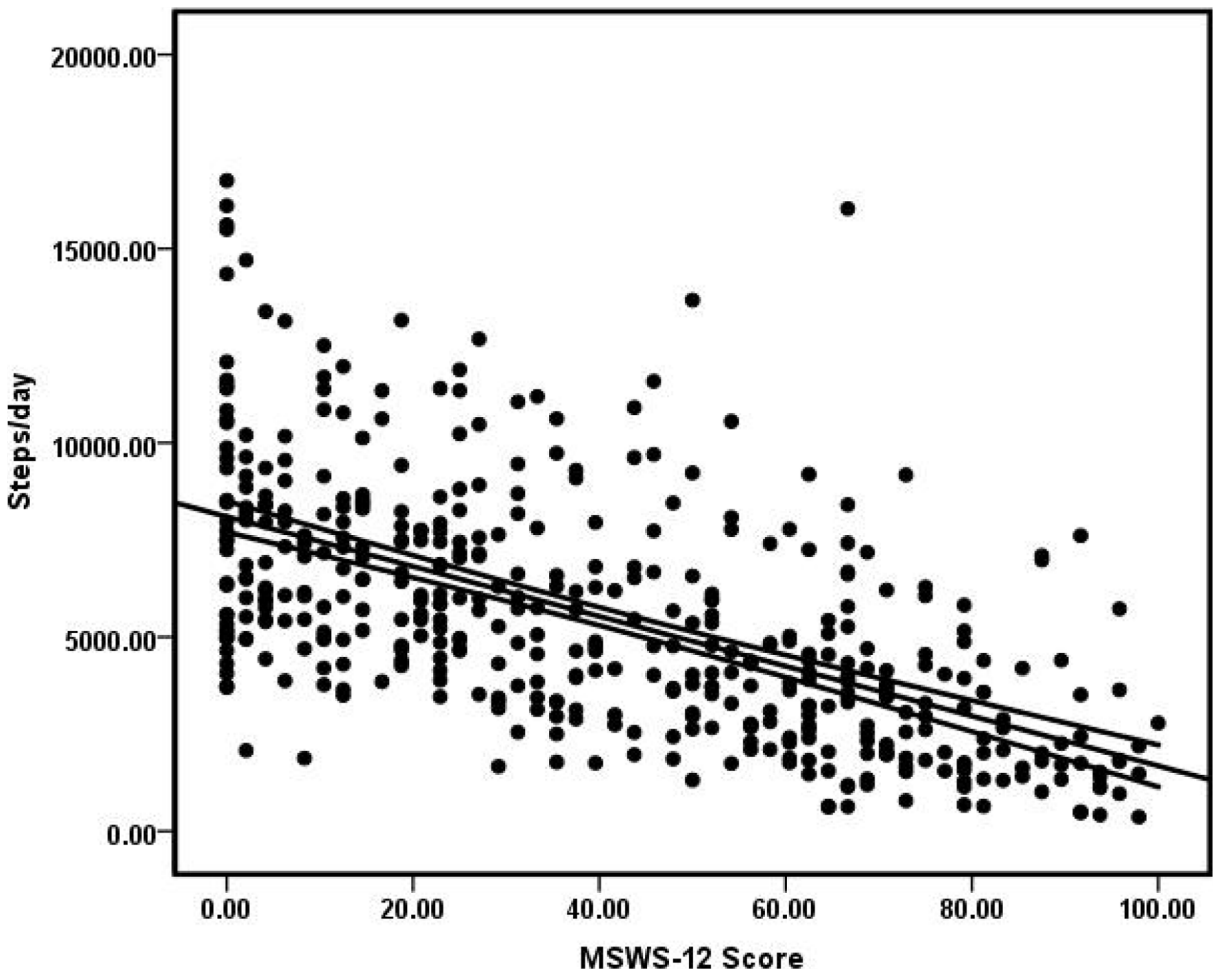
Scatter plot of the association between Multiple Sclerosis Walking Scale-12 (MSWS-12) scores and steps/day in persons with multiple sclerosis.

**Figure 2:**
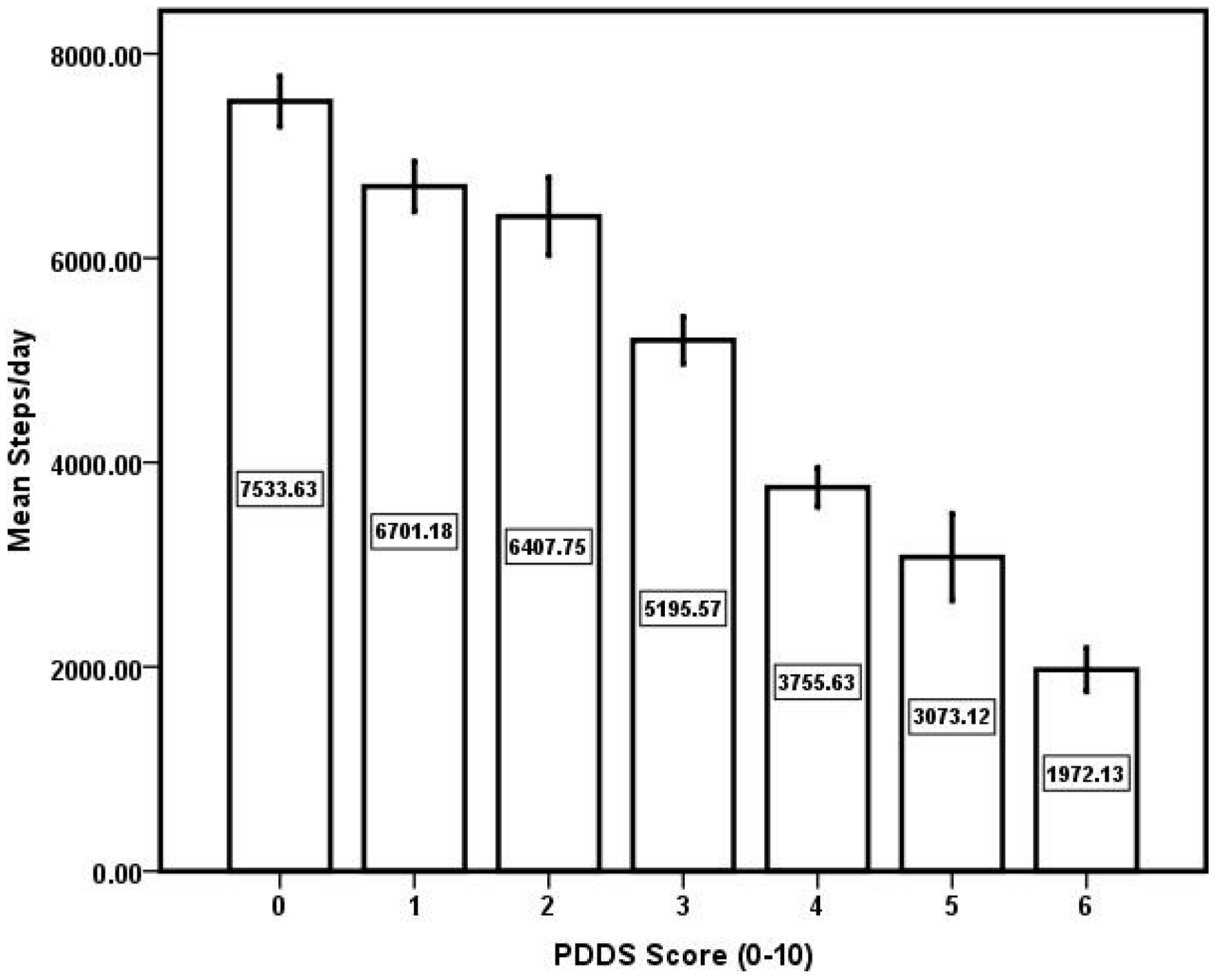
Bar graph of the association between Patient Determined Disease Steps (PDDS) scale scores and steps/day in persons with multiple sclerosis. The number within the bars represents the mean score for steps/day per level of the PDDS.

We regressed steps/day on MSWS-12 scores and the association was presented as a scatter plot in Figure 1. The regression model was statistically significant (*F_1,455_* = 241.68, *p* = .0001) and MSWS-12 scores accounted for 35% of the variance in steps/day (Adjusted *R* ^2^ = .35). The resulting regression equation of steps/day  = 8,100–64.2×MSWS-12 score indicated that every 10-point increase in MSWS-12 scores yielded a reduction of 642 steps/day. We then regressed steps/day on PDDS scores and the association was presented as a bar graph in Figure 2. The regression model was statistically significant (*F_1,588_* = 255.74, *p* = .0001) and PDDS scores accounted for 30% of the variance in steps/day (Adjusted *R* ^2^ = .30). The resulting regression equation of steps/day  = 7680–915×PDDS score indicated that every 1-point increase in PDDS scores yielded a reduction of 915 steps/day. Overall, the mean MCID across the two self-report scales that capture subtle, perceived changes in ambulation was ∼779 steps/day (range  = 642–915) or 14% of the mean steps/day for the MS sample.

### Analysis of variance

#### Steps/day Based on Clinical Characteristics and Health Outcomes in Persons with MS

**Figure 3:**
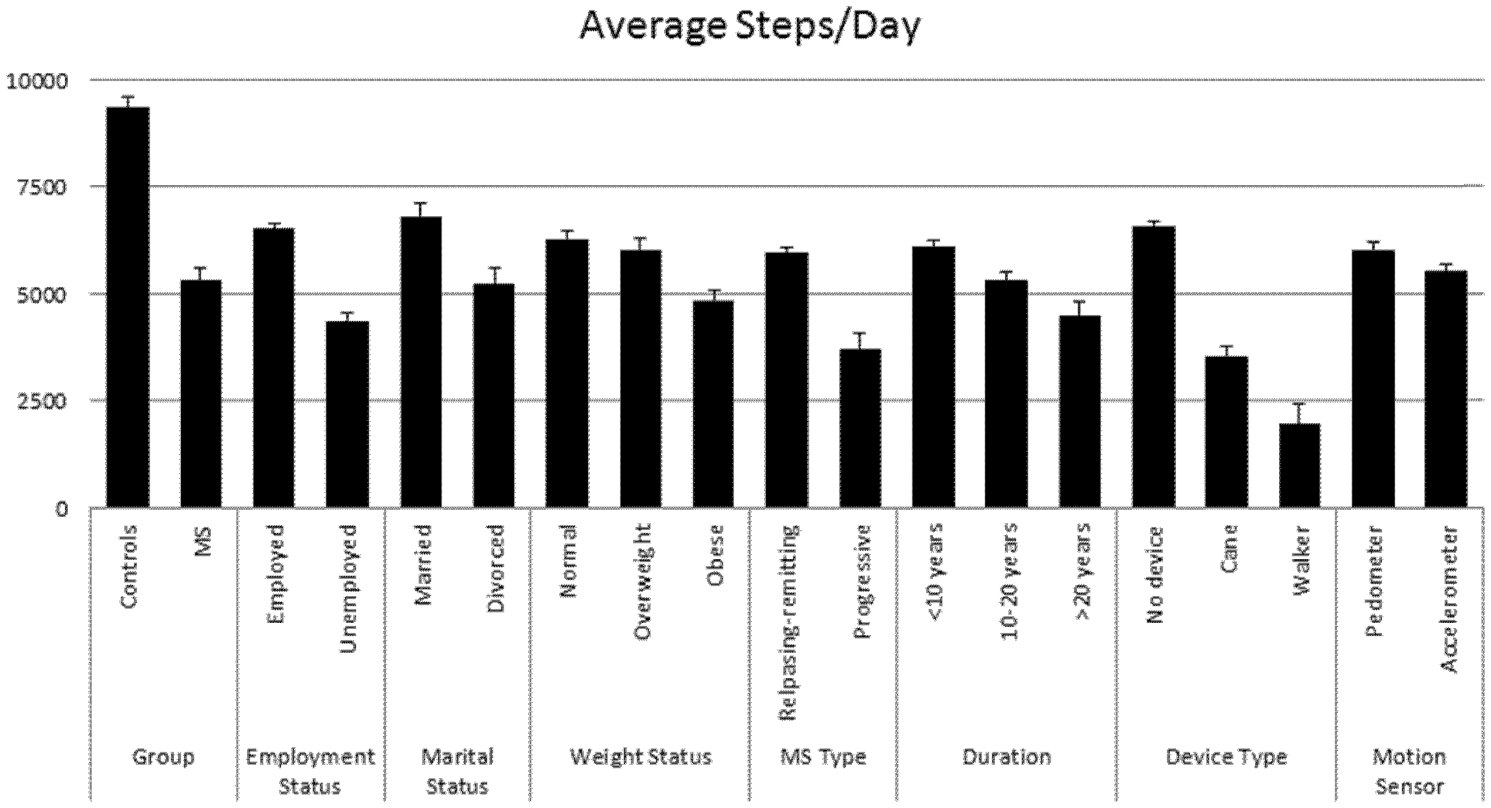
Graphical representation of steps/day by MS and control groups and by real-life, health, clinical, and motion sensor characteristics among only those with multiple sclerosis.

There were statistically significant differences in steps/day among persons with MS when considering MS type (*F*_1,766_ = 37.67, *p* = .0001), disease duration (*F*_1,773_ = 12.60, *p* = .0001), and weight status (*F*_1,521_ = 10.95, *p* = .0001); the mean scores are provided in Table 2 and displayed in Figure 3. When comparing clinical course of MS (i.e. relapsing-remitting vs. progressive MS), the difference was 2,233 steps/day (95% *CI* = 1,518–2,947) and moderate-to-large in magnitude (*d* = 0.73). When consider disease duration, the difference between short (i.e., <10 years) and long disease duration was 788 steps/day (95% *CI* = 166–1,411) and small in magnitude (*d* = 0.25), whilst between middle and long disease duration the difference was 1,621 steps/day (95% *CI* = 765–2,476) and moderate in magnitude (*d* = 0.52). There was no significant difference in steps/day between middle (i.e., 10–20 years) and longer (i.e., >20 years) disease duration. Regarding weight status, there was no significant difference in steps/day between normal and overweight groups. By comparison, there were statistically significant differences of 1,436 steps/day (95% *CI* = 676–2,196) and 1,196 steps/day (95% *CI* = 356–2,036) that were moderate in magnitude when comparing normal vs. obese (*d* = 0.49) and overweight vs. obese (*d* = 0.43) persons with MS, respectively. Overall, the mean MCID across clinical characteristics and health outcomes that demonstrated statistically significant differences between groups was ∼1,455 steps/day (range  = 788–2,233) or 26% of the mean steps/day for the MS sample.

**Table 2:** Steps/day in persons with multiple sclerosis by clinical, health, and real-life characteristics.

Category	Groups	*N*	Mean	*SD*	95% *CI*
MS type	Relapsing- remitting MS Progressive MS	68979	59513718	31312382	5722, 61803042, 4395
Disease duration	≤10 years10–20 years≥20 years	49019888	609353054472	322729322519	5820, 63664875, 57343827, 5117
Weight status	NormalOverweightObese	226146152	626460244828	326731212483	5870, 66595533, 65154347, 5309
Employment status	EmployedUnemployed	485289	65104361	31762536	6247, 67744020, 4702
Marital status	MarriedDivorced	121104	67975240	38422822	6187, 74084581, 5899
Device type	No deviceCaneWalker	42712934	656835391972	292021701260	6311, 68253071, 40061062, 2882

*Note*. SD  =  standard deviation. CI  =  confidence interval represented as upper and lower boundaries. MS  =  multiple sclerosis. The sample sizes per characteristic in the MS sample differ because of missing data or focus on specific levels of a variable.

#### Steps/day Based on Real-life Anchors in Persons with MS

There were statistically significant differences among persons with MS as a function of employment (*F*_1,772_ = 95.90, *p* = .0001), marital (*F*_1,223_ = 11.67, *p* = .0001), and device type (*F*_1,587_ = 95.49, *p* = .0001); the mean scores are provided in Table 2 and displayed in Figure 3. When considering the employment status of persons with MS (i.e., employed vs. unemployed), the mean difference was 2,150 steps/day (95% *CI* = 1,719–2,580) and moderate-to-large in magnitude (*d* = 0.73). Analysis of marital status (married vs. divorced) indicated a difference of 1,557 steps/day (95% *CI* = 659–2,456) that was moderate in magnitude (*d* = 0.46). The differences were 3,029 steps/day (95% *CI* = 2,377–3,681) and 4,596 steps/day (95% *CI* = 3,440–5,752) and large in magnitude when considering device type as no device (none; PDDS score  = 1–3) vs. cane (PDDS score  = 4–5; *d* = 1.10) and no device (none) vs. walker (PDDS score  = 6; *d* = 1.64), respectively, among persons with MS. Comparing device type categorized as cane vs. walker, the difference was 1,567 steps/day (95% *CI* = 316–2,817) and moderate in magnitude (*d* = 0.79). Overall, the mean MCID across real-life anchors that demonstrated statistically significant differences between groups was ∼2,580 steps/day (range = 1557–4596) or 45% of the mean steps/day for the MS sample.

#### Steps/day between MS and Controls

There was a statistically significant and large (*F*_1,941_ = 167.87, *p* = .0001, *d* = 1.13) difference of 3,640 steps/day (95% *CI* = 3,088–4,191) between persons with MS and controls (i.e., 64% of the mean steps/day for the MS sample). The mean for the controls (*N* = 157) was 9,342 steps/day (*SD* = 3,588, 95% *CI* = 8,838–9,845), whereas the mean for the persons with MS (*N* = 786) was 5,702 steps/day (*SD* = 3,134, 95% *CI* = 5,477–5,927). This difference was 2,747 steps/day (95% *CI* = 2,151–3,344), after controlling for age, BMI, sex, employment, education, income, and race as covariates (*p* = .0001, *d* = 0.88), or 48% of the mean steps/day for the MS sample. There further was a statistically significant (*F*_1,743_ = 121.69, *p* = .0001) difference in steps/day between PDDS groups (1–3, 4–5, and 6) and healthy controls. The mean for the controls (*N* = 157) again was 9,342 steps/day (*SD* = 3,588, 95% *CI* = 8,838–9,845), whereas the means for the persons with MS were 6,568 steps/day (*SD* = 2,920, 95% *CI* = 6,311–6,825), 3,539 steps/day (*SD* = 2,170, 95% *CI* = 3,071–4,006), and 1,972 steps/day (*SD* = 1,260, 95% *CI* = 1062–2,882) per PDDS group (1–3, 4–5, and 6, respectively).

#### Steps/day Based on Motion Sensor Type in Persons with MS

There was not a statistically significant difference in steps/day among persons with MS when considering the type of motion sensor (*F*_1,784_ = 3.97, *p*>.01). The mean for the pedometer (*N* = 273) was 6,007 steps/day (*SD* = 3,277, 95% *CI* = 5,635–6,379), whereas the mean for the accelerometer (*N* = 513) was 5,540 steps/day (*SD* = 3,045, 95% *CI* = 5,269–5,811). The difference between motion sensors was 467 steps/day (95% *CI* = 7–927; 9% of the mean steps/day for the MS sample) and small in magnitude (*d* = 0.15). This indicated that the MCID estimates did not dependent on the type of motion sensor as there was no difference in steps/day between motion sensors. Such a conclusion was confirmed in additional analyses generating MCID estimates using only accelerometer derived steps/day as such estimates differed marginally from those reporting using both pedometers and accelerometers.

## Discussion

This study estimated values for the clinical importance (i.e., MCID) of steps/day as a measure of free-living walking behavior in persons with MS. To that end, the MCID for steps/day ranged between 779 and 2,747 steps/day based on stages of expressing the developing impact of MS with the smallest value reflecting subtle, perceived differences in ambulation by the patient and the largest value reflective the cumulative impact of MS compared with healthy controls. These MCIDs translate into a range of difference between 14% and 48% of the mean steps/day for the entire MS sample. Such vantage points suggest that a value of ∼800 steps/day (∼15%) would reflect the smallest clinically important change in community-based walking behavior in persons with MS (corresponding with incremental, unit changes in perceived walking impairment or disability identified by the patient), whereas a value of ∼2,750 steps/day (∼50%) would reflect the largest clinically important change in community-based walking behavior (corresponding with global burden of MS compared against controls without the disease). We do not have data regarding the MCID values associated with a relapse. Nevertheless, these MCID estimates for steps/day could be applied in isolation or in combination with those developed for the MSIS-29 physical subscale (i.e., 8 point change) [16] and the MSWS-12 (i.e., 4–6 point change) [17] in therapeutic interventions among persons with MS. The combined approach might provide a comprehensive assessment of clinically important change among persons with MS, and likely depends on the focus and intended outcomes of a clinical trial.

An important consideration is the attainability of the MCID values of 800 to 2,750 steps/day in clinical research involving persons with MS. This might be addressed based on physical activity interventions for increasing daily ambulatory activity in persons with MS; no interventions of disease modifying agents have examined steps/day as an outcome. To that end, physical activity interventions lasting 12 weeks have reported changes of between ∼1,400 and ∼1,800 steps/day [18], [19]. Such changes are larger than the smallest MCID of ∼800 steps/day identified in the current analysis and fall in the middle of the range of MCID values. This would support the idea that such MCID values for steps/day are attainable in clinical research involving persons with MS who take part in a physical activity intervention, and perhaps other therapeutic interventions. Importantly, we are aware of one study that reported a change of ∼600 steps/day one month after treatment for a relapse using intravenous methylprednisolone therapy in 49 consecutive patients with RRMS [20].

This study provides information on the clinical importance of steps/day in persons with MS. Such a contribution extends the research on validity of motion sensor output, particularly steps/day, as a measure of free-living walking behavior in MS [2]. These results might set the stage for inclusion of motion sensors in interventional trials measuring ambulation. The inclusion of motion sensors might provide information about changes in free-living walking behavior (i.e., improvement and worsening). To date, we are unaware of research that has adopted motion sensors and capitalized on such an opportunity for clinical trials in MS.

There are limitations of this study that should be considered when interpreting the results. One limitation is that the sample primarily consisted of women with relapsing-remitting MS and this might limit generalizability and applicability among men and those with a progressive course of MS. Consequently, the MCID may differ for another MS cohort with different demographics, including level of disability. The methods for identifying clinical importance of steps/day involved the reliance upon self-reported scales and data, rather than a physician-based rating or performance outcomes. This reliance upon self-report for classification of clinical and health variables could introduce error that biases the estimates of the MCIDs. The third limitation is that we did not compare differences in steps/day between demographic or health variables across the group factor of MS and controls (i.e., an interaction of the characteristic by MS vs. control groups), as there were not sufficient numbers of cases per level of the variables in the healthy controls (e.g., there were only nine unemployed controls). This does not permit an examination of the generalizability or specificity of the differences in steps/day per real-life (e.g., unemployment) and health (e.g., overweight and obese weight status) variables. This further does not permit an understanding of variables such as weight status as antecedents or consequences of reductions in steps/day. Lastly, the MS and control groups differed in sociodemographic variables, and we controlled for such differences in the analysis, but there may be other variables besides MS and its manifestations that account for differences between MS and healthy controls.

Overall, this secondary analysis established MCID estimates for understanding the clinical importance of steps/day as a measure of free-living walking behavior in MS. These data along with existing evidence for reliability and validity will be important for designing clinical trials involving motion sensors and steps/day as an outcome measure of free-living walking behavior in MS. Such results will guide and inform researchers and clinicians on the clinical importance of steps/day in persons with MS.
